# Living on the Edge: Juvenile Foraging and Adult Breeding Ecology of an Aerial Top Predator in Urban Environments

**DOI:** 10.1002/ece3.73097

**Published:** 2026-02-11

**Authors:** Marc Engler, Oliver Krone

**Affiliations:** ^1^ NABU Landesverband Berlin e.V Berlin Germany; ^2^ Department of Wildlife Diseases Leibniz Institute for Zoo and Wildlife Research Berlin Germany

**Keywords:** *Buteo buteo*, Common Buzzard, GPS telemetry, habitat use, raptor biology, species distribution

## Abstract

Urbanization is irreversibly transforming natural ecosystems globally, yet we still lack a holistic understanding of the challenges and opportunities that urban environments present to wildlife. To investigate how raptors utilize urban landscapes, we analyzed 4 years of GPS tracking data of 22 juveniles collected during natal dispersal, combined with 7 years of nest‐monitoring data from an urban population of the Common Buzzard ( *Buteo buteo* ), an opportunistic rodent specialist, in Berlin (Germany). Our goals were threefold: (a) to estimate core foraging ranges (CFRs), (b) to characterize small‐scale temporal foraging areas (TFAs) within these ranges, and (c) to model the breeding habitat suitability based on environmental predictors. CFRs were distributed across the city, with a mean area of 11.0 km^2^ (±13.9 sd). TFAs within these ranges had a mean area of 0.2 km^2^ (±0.3) and were occupied over a mean time span of 274 days (±198). TFAs were mostly located in residential open areas (e.g., courtyards) and were characterized by decreasing area and shorter occupation duration with increasing human population density. Urban TFAs showed a high prevalence of rats ( *Rattus norvegicus* ). Active pest control measures were observed at most TFAs, particularly in residential areas. We mapped a total of 17.3 km^2^ of suitable breeding habitat within the study area (217 km^2^) and 45.4 km^2^ when projected across the city region (890 km^2^). Suitable breeding habitat consisted of isolated patches, i.e., single courtyards in residential areas, and a network of urban green spaces, e.g., city parks and cemeteries. Cities can provide profitable foraging and breeding conditions for raptors, with abundant prey in proximity to suitable breeding sites. The study improves our understanding of how raptors utilize the urban city landscape and underlines the importance of urban green spaces as a basis for the protection and conservation of urban wildlife.

## Graphical abstract

We analyzed a combination of GPS tracking data and nest‐monitoring data from an urban population of an opportunistic rodent specialist, the Common Buzzard ( *Buteo buteo* ), to analyze how raptors utilize urban landscapes. Buzzards occupied even extreme habitats such as residential areas for foraging and breeding and benefit from lucrative foraging conditions, abundant prey and mature tree stands. The advantages may be counteracted by the risk of poisoning with anticoagulant rodenticides used for pest control, thus underlining the thin line between chances and risks for urban wildlife in cities.

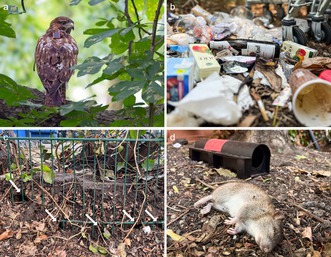

## 1 Introduction

At present, approximately 57% of the global human population lives in cities and by 2040, this figure is projected to reach almost two‐thirds of the world's population (United Nations, 2023). The accelerated expansion of urban areas and the transformation of land for human use have inevitable impacts on wildlife, presenting new opportunities and major challenges. The ability of species to cope with and adapt to such novel environments in proximity to humans is a determining factor in their habitat selection (Kark et al. 2007). In urban areas, avian predators may thrive on the availability of attractive nest sites (Møller et al. 2012) as well as the year‐round availability and high abundance of profitable prey, including small mammals (e.g., rats, mice and squirrels) or various bird species (Sumasgutner et al. 2014; Zúñiga‐Vega et al. 2023).

Multiple raptor species have successfully colonized urban environments across the globe, including the Northern Goshawk ( *Accipiter gentilis* ; Engler et al. 2021; Merling de Chapa et al. 2020) and the Eurasian Kestrel (*Falco tinnunculus*, Kettel et al. 2018; Sumasgutner et al. 2014, 2023) in Europe, the African Crowned Eagle ( *Stephanoaetus coronatus* ) in South Africa (Muller et al. 2020), the Red‐tailed Hawk ( *Buteo jamaicensis* ) across North America (Boal and Dykstra 2018; Cooper et al. 2021) and the Red‐necked Falcon ( *Falco chicquera* ) in Asia (Foysal and Panter 2024). In such areas, raptors with behavioral plasticity and tolerance for humans may thrive where nesting opportunities are available, often in proximity to foraging spots with high prey availability (Kettel et al. 2018; Møller and Díaz 2018; Reynolds et al. 2019). In general, smaller raptor species exhibit greater tolerance and are more abundant in cities than their larger counterparts (Cooper et al. 2022; Headland et al. 2023). Survival and reproductive success may also be affected by hunting behavior and prey abundance; avian specialists such as Peregrine Falcons ( *Falco peregrinus* ) exhibit higher breeding success in cities than typical rodent hunters such as Eurasian Kestrels, which can suffer from lower breeding success in urban centres (Kettel et al. 2019; Sumasgutner et al. 2014).

Although juvenile dispersal constitutes a key life‐history phase, research on urban raptor populations is often constrained by logistical challenges and limited opportunities for long‐term tracking. During this life‐history stage, juveniles develop foraging proficiency and acquire spatial knowledge that may influence their survival and later settlement decisions (Morrison and Wood 2009; Serrano 2018). Because juvenile raptors are unconstrained by breeding duties, their movements provide unique insights into habitat use and foraging decisions that cannot be inferred from breeding adults alone (García‐Macía et al. 2023). Thus, information on juvenile dispersal is valuable for understanding population dynamics in birds of prey, particularly in novel, human‐dominated landscapes (Delgado and Penteriani 2008).

Concurrently, urban environments may expose raptors to anthropogenic threats, including the risk of collision with windows, trains, and vehicles (Cusa et al. 2015; Walls and Kenward 2020; Bullock et al. 2024). Furthermore, raptors may experience an elevated prevalence of diseases (Krone et al. 2006; Merling de Chapa et al. 2021) and intoxication (Badry et al. 2020, 2021).

Particularly for rodent specialists, the use of anticoagulant rodenticides (ARs) to control rat populations in urban areas poses a substantial risk (Elliott et al. 2016; Gomez et al. 2022; Murray 2020). Commensal rat species like the Brown Rat ( *Rattus norvegicus* ) occur almost globally and benefit from human refuse, shelter, and reduced predation risk in urban environments (Richardson et al. 2025; Shukla and Wilmers 2024). Where rats are particularly abundant and cause damage to infrastructure in urban environments, local populations are often managed via baiting boxes containing anticoagulant compounds (Jacob and Buckle 2018). Since rats constitute a highly profitable and abundant prey species for both rodent‐specialist and generalist predators (Torre et al. 2024; Walls and Kenward 2020), raptors may be exposed to increased levels of ARs through secondary poisoning (López‐Perea and Mateo 2018). The raptor species most at risk include those that can adapt to human‐modified environments and those that feed primarily by scavenging, such as the Common Buzzard ( *Buteo buteo* ).

The Common Buzzard is considered one of the most common diurnal raptor species in Europe, with an estimated population size of 0.9–1.2 million pairs (BirdLife International 2021). It is thus a suitable model organism for studies addressing dispersal, population demography, and habitat use (Schindler et al. 2012; Walls and Kenward 1995, 1998, 2020). Common Buzzards primarily utilize a sit‐and‐wait hunting strategy along the edges of open areas such as meadows, fields, and clear‐cut forest patches, which are known to be rich in rodents. Mature tree stands and forest patches in proximity to foraging grounds are frequently utilized for nest building (Newton 1979). Despite its abundance, the Common Buzzard is rarely associated with urban environments (Kettel et al. 2018; Walls and Kenward 2020). The German capital of Berlin is one of the only metropolises worldwide with a known established population of this species within the core city area.

The improvement in tracking device technology has led to an exponentially increasing output of high‐resolution tracking data, expanding the spatio‐temporal scale at which movement patterns can be analyzed (Hooten et al. 2017; Kays et al. 2015). GPS tracking data thus constitutes an important resource for studying movements of dispersing juveniles and identifying key areas relevant for conservation and management, particularly in heterogeneous and human‐dominated landscapes.

A comprehensive understanding of how Common Buzzards utilize urban environments is essential for understanding why some species adapt to anthropogenically shaped environments, while others do not (Lowry et al. 2013). In this paper, we aimed to examine how Common Buzzards utilize the urban landscape by investigating the spatio‐temporal patterns of both foraging areas and breeding habitats in a well‐monitored urban population. Thus, we analyzed an extensive dataset of GPS tracking data from juveniles collected during natal dispersal as well as long‐term nest‐monitoring data of breeding pairs asking the following research questions: First, which areas do Common Buzzards occupy as core foraging ranges during natal dispersal in the city area? Second, how are specific foraging hotspots within these ranges characterized with respect to the environmental composition, i.e., habitat type, prey availability, and pest control measures? Third, to what extent is breeding habitat suitability shaped by habitat composition, habitat structure, and human impact? Where and in what quantities is suitable breeding habitat available in the heterogeneous city environment?

We hypothesized that juvenile Common Buzzards exploit temporarily available and local food sources in the city area, including foraging sites in proximity to human activity, such as residential and recreational areas. We hypothesized that at these locations, rats are abundant and that active pest control measures are present. Conversely, we hypothesized that Common Buzzards utilize green habitat islands that feature reasonable tree stands and minimal to moderate human presence for breeding, thus preferring areas such as city parks and cemeteries over residential areas with higher levels of human‐related disturbance.

## 2 Methods

### 2.1 Study Area

The study area was located in the city centre of the federal state of Berlin in North‐East Germany, covering an area of approximately 217 km^2^ (Figure 2a). The human population of Berlin, the state capital, is estimated to exceed 3.6 million people, inhabiting an area of approximately 890 km^2^. Although almost 60% of the city surface is sealed with housing and traffic, more than 2.500 city parks and 220 cemeteries are distributed throughout the city area. The city centre covers an area of approximately 200 km^2^ and is characterized by a high degree of urbanization, including high‐rise buildings, sealed artificial surfaces, and considerable human impact in terms of human presence and population density (Figure 2c–f). Along a gradient from the city centre towards the outskirts, single‐family housing areas with gardens, as well as forest patches and lakes characterize a suburban transition. In the urban periphery, approximately 160 km^2^ of forest contribute to the city's network of green spaces. Berlin is an urban island fully surrounded by the federal state of Brandenburg, which is dominated by agricultural land (48%) and forest patches (36%, www.statistik‐berlin‐brandenburg.de, 2020).

### 2.2 Foraging Ecology of Juveniles

#### 2.2.1 GPS Tracking

We analyzed movement data of 22 juvenile Common Buzzards from urban breeding pairs in Berlin, collected during natal dispersal over a study period of four years from 2022 to 2025. Juveniles were equipped with solar‐powered GPS‐GSM OrniTrack‐25 transmitters (Ornitela, Vilnius, Lithuania, 25 g) as nestlings at an age between 32 and 40 days, using a backpack‐style harness made from 6‐mm‐wide Teflon ribbon tubes. Transmitters, including harness, weighed approximately 27 g (< 3% of the birds' body weight, Kenward et al. 2001).

GPS trackers were programmed to record GPS locations at intervals ranging from once every 5 min to once every 4 h between 04:00 and 22:00 local time (Central European Summer Time, CEST; i.e., UTC + 2), depending on the season and light conditions (e.g., low energy supply during winter months). The horizontal position errors of trackers averaged < 3 m. We omitted particularly imprecise fixes based on horizontal dilution of precision (fixes with HDOP > 4 or n_satellites_ < 5, Gupte et al. 2022).

Since we were interested in analyzing core foraging ranges of juveniles during natal dispersal, we limited our analyses to GPS locations collected after the onset of natal dispersal (thus omitting exploratory movements after fledging) and until an age of two years (≤ 730 days), when Common Buzzards typically end the natal dispersal phase and attempt their first breeding (Walls and Kenward 2020). We determined the onset of natal dispersal using the distance threshold method, which has been demonstrated to be particularly effective for raptor species with a distinct event of emigration, that is, a rapid increase in distance to the parental territory (Engler and Krone 2023; Weston et al. 2013). We considered the onset of natal dispersal as the first day outside the fixed circular parental territory, with no return within the following seven days. Given the absence of reliable data concerning the territorial size of urban breeding pairs, we assumed a parental home range size of 3.1 km^2^ (radius of 1000 m) based on reviewed home range sizes of Common Buzzards in Europe (Walls and Kenward 2020). We selected a temporal threshold of seven days, because we considered this a realistic period for juvenile Common Buzzards to return from exploratory movements without any foraging success and yet no serious impact on their physiology (Engler and Krone 2021; Walls and Kenward 2020).

As our study focused on foraging patterns of a strictly diurnal raptor and occurred within the city area of Berlin, we filtered the data set to include only GPS positions within the city area of Berlin and GPS positions collected only during daytime, including nautical twilight, based on the day of the year and exact location using the R package *suncalc* (Thieurmel and Elmarhraoui 2022). Lastly, we only considered individuals for which tracking data were available over a minimal period of 30 tracking days in order to adequately reflect individual movements. The resulting data set used to estimate core ranges and temporal foraging areas consisted of 120.431 GPS positions of 22 juveniles (see Supporting Information S1).

All animal handling procedures adhered to protocols no. 0144/21, as approved by the Berlin State Office for Health and Social Affairs (LAGeSo), and no. III B 294—OA‐AS/FAS/693, as approved by the Senate Department for Urban Mobility, Transport, Climate Action, and the Environment.

#### 2.2.2 Estimation of Core Foraging Ranges

We conducted a three‐step analysis to identify and characterize foraging areas utilized by Common Buzzards during natal dispersal and within the city area: (a) estimation of individual‐level 50% “core foraging ranges” (hereafter CFRs), (b) identification of more detailed “temporal foraging areas” (hereafter TFAs) as hotspots located within the CFRs, and (c) characterization of TFAs with respect to environmental properties, addressing habitat type, prey availability, and human disturbance through in‐field observations.

We implemented Autocorrelated Kernel Density Estimations (AKDE, Fleming et al. 2015) as a state‐of‐the‐art range estimation tool to fit continuous time stochastic process movement models for each individual using the *ctmm* package (Fleming and Calabrese 2023), following guidelines provided by Silva et al. (2022). Although AKDE are generally applied to estimate the “home range” sizes of individuals, in this study we tracked juvenile Common Buzzards during natal dispersal, and thus at a life‐history phase in which they have not yet established their own territories (Walls and Kenward 2020). The prevailing concept defines the term “home range” as an area that is traversed by an individual in its normal activities of food gathering, mating, and caring for young. As this definition does not meet the particular life‐history phase of natal dispersal (Burt 1943), we rephrased the term to “core foraging range” based on other movement ecology studies (Bryan et al. 2012; Powell and Mitchell 2012; Seaman and Powell 1996). We thus opted for a term that captures both the ranging character of dispersal movements as well as opportunistic foraging as the prevailing behavior displayed by juvenile raptors during natal dispersal (Kenward et al. 1993; Walls and Kenward 2020).

**FIGURE 1:**
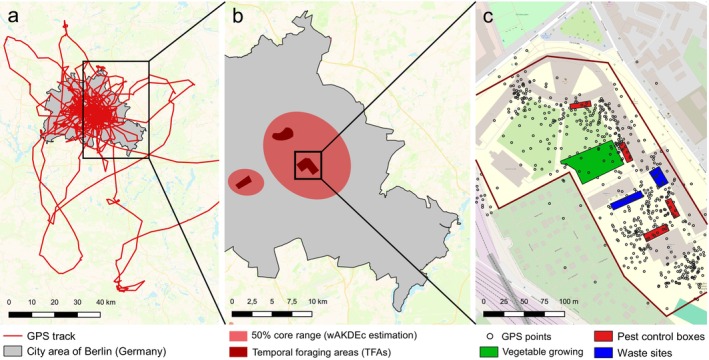
Schematic workflow of the estimation of core foraging ranges (CFRs) and characterization of urban temporal foraging areas (TFAs) of juvenile Common Buzzards. (a) GPS track of one individual in and around the city area (gray) of Berlin (Germany). (b) 50% core foraging range contour lines (red) estimated by wAKDEc for the same individual. Dark red polygons indicate single TFAs as clusters of GPS points within the 50% core range, representing local foraging hotspots. (c) Visualization of in‐field records of variables of interest at one exemplary foraging hotspot (black box in b). Variables include multiple features promoting the presence of rats (e.g., vegetable growing, public waste), direct indication of vole or rat presence or active pest control measures (Table 1). The base map (data from OpenStreetMap, under ODbL) displays buildings in brown–gray, open sealed areas in beige, and grass patches in green. All maps are north‐oriented.

We checked for range residency using the variogram analysis provided by the *ctmm* package to ensure individual ranges were no longer expanding. We then implemented area corrected and weighted AKDEs (wAKDEc), both to account for overestimation of the core foraging range with the Gaussian reference function and to correct for irregular sampling schedules and missing data, which was the case for most of the tracked Common Buzzards (Seaman and Powell 1996). We used perturbative hybrid residual maximum likelihood (pHREML) estimation, due to its extensive applicability and the integration of the benefits of stability and bias correction when employing machine learning (ML) or REML estimation (Silva et al. 2022). For each individual, we fitted multiple candidate movement models and identified the model best fitting the autocorrelation structure via the AICc (Fleming et al. 2015; Fleming and Calabrese 2017, see Supporting Information S2). Subsequently, we extracted individual 50% core foraging range contour lines based on the best fitting movement model to represent regions of most concentrated activity and foraging effort for further analysis (Figure 1b; Fleming and Calabrese 2017; Seaman and Powell 1996; Worton 1989).

#### 2.2.3 Characterization of Temporal Foraging Areas

As a second step, we characterized the environmental composition and spatio‐temporal extent of TFAs established in the city area. We defined TFAs as isolated and spatially restricted clusters of GPS points within the broader CFRs (Figure 1b). For each individual, we generated TFAs as 95% minimum convex polygons around all GPS points within a homogeneous type of habitat, e.g., a single city park. We only considered clusters with a minimum of 30 GPS positions over a minimum period of 10 days (i.e., a minimum of 3 GPS points per day).

Subsequently, we comprehensively characterized TFAs through in‐field visits with the objective of documenting the prevailing local foraging conditions. We addressed the following qualitative properties: (a) the general environment, specifically the habitat type and level of human presence; (b) the presence of voles as available natural prey; (c) the presence of rats as human‐promoted prey or anthropogenic features that promote their presence; and (d) active pest control measures in the form of AR application through baiting boxes (Figure 1c; see Table 1 for details). During the period when juveniles occupied the areas, each TFA was surveyed once at dawn, once at midday (11:00–15:00), and once at dusk during the same or consecutive days. We scanned the entire area of TFAs for the presence of voles or rats, which included any observations of active burrows, feces, or direct observation of animals. Likewise, we protocolled any human features promoting the presence of voles or rats, including excessive public waste around waste disposal areas or excessive bird feeding on the ground (Table 1). Lastly, we protocolled whether any pest control measures were active (i.e., baited pest control boxes with up‐to‐date labels) during visits. Since permanent baiting is prohibited under the EU Biocidal Regulation (EU 528/2012), active baiting boxes are indicative of an ongoing local rat infestation. In only two rare cases, we found inactive baiting boxes without labels and baits, and thus did not consider them in the survey.

**TABLE 1:** Variables collected during in‐field visits characterizing urban temporal foraging areas (TFAs) of tracked juvenile Common Buzzards during natal dispersal.

Type	Variable	Description
Environment	Habitat type (qualitative, 8 levels)	Forest, highway/railway (infrastructure), allotment, cemetery, city park, depot, recreational areas, residential areas
	Human presence (qualitative, 3 levels)	Low (e.g., forest, highway/railway, allotment), moderate (e.g., cemetery, city park, depot), high (e.g., recreational areas, residential areas)
Prey availability	Human features promoting presence of rats (0/1)	Vegetable growing, public waste, excessive wildlife feeding
	Indication of presence of voles (0/1)	Observation of burrows, feces; sightings
	Indication of presence of rats (0/1)	Observation of burrows, feces; sightings
Human impact	Pest control (0/1)	Observation of active pest control boxes

*Note:* all variables were qualitative (i.e., classified in levels or collected as presence/absence of the variables addressing prey availability or human impact).

To assess the spatio‐temporal extent of foraging, we calculated the area of individual TFAs as well as the time span over which they were frequently visited. As the tracking data exhibited periods of missing data for most individuals, particularly during the winter months, it was not possible to distinguish between periods of true presence and absence. Consequently, an analysis of the data at the level of individual visits to TFAs (i.e., temporal nichening) was not feasible. Instead, as an approximated measure for the temporal extent of TFA usage, we calculated the total time span over which a TFA was visited as the 95% quantile between the first and last GPS location at a given TFA, thus accounting for outliers.

#### 2.2.4 Statistical Analysis

To investigate whether the temporal and spatial extent of TFAs was related to the degree of human presence, we fitted two separate generalized linear mixed‐effects models (GLMM). These predicted the time span of occupation (in days) across TFAs as well as the utilized area (in ha) as the response variable, based on the average human population density (inhabitants/ha) as the predictor variable, which was obtained from the Copernicus online database (https://land.copernicus.eu) at a resolution of 100 m. To model the time span of occupation, we fitted a GLMM with a Poisson error distribution and log link function. To model the utilized area across TFAs, we fitted a GLMM with a Gaussian error distribution on log‐transformed response and predictor variables. In both models, we accounted for variation among individuals by integrating the individual ID as a random effect (Rousset and Ferdy 2014). For model building, we used the function *fitme* from the R package *spaMM* version 4.5.0, while setting the fitting method to “PQL/L.” We checked model assumptions through residual plots simulated via parametric bootstrapping using the R package *DHARMa* version 0.4.6 (Hartig 2022). We tested the significance of variables using parametric bootstraps via the function *LRT* from *spaMM* based on 1000 bootstrap replicates to build the distribution of the test statistic of each Likelihood Ratio Test (LRT) under the null hypothesis. All data processing and statistical analyses were all performed in R version 4.5.1 (R Core Team 2024).

### 2.3 Breeding Ecology of Adults

#### 2.3.1 Breeding Site Selection

**FIGURE 2:**
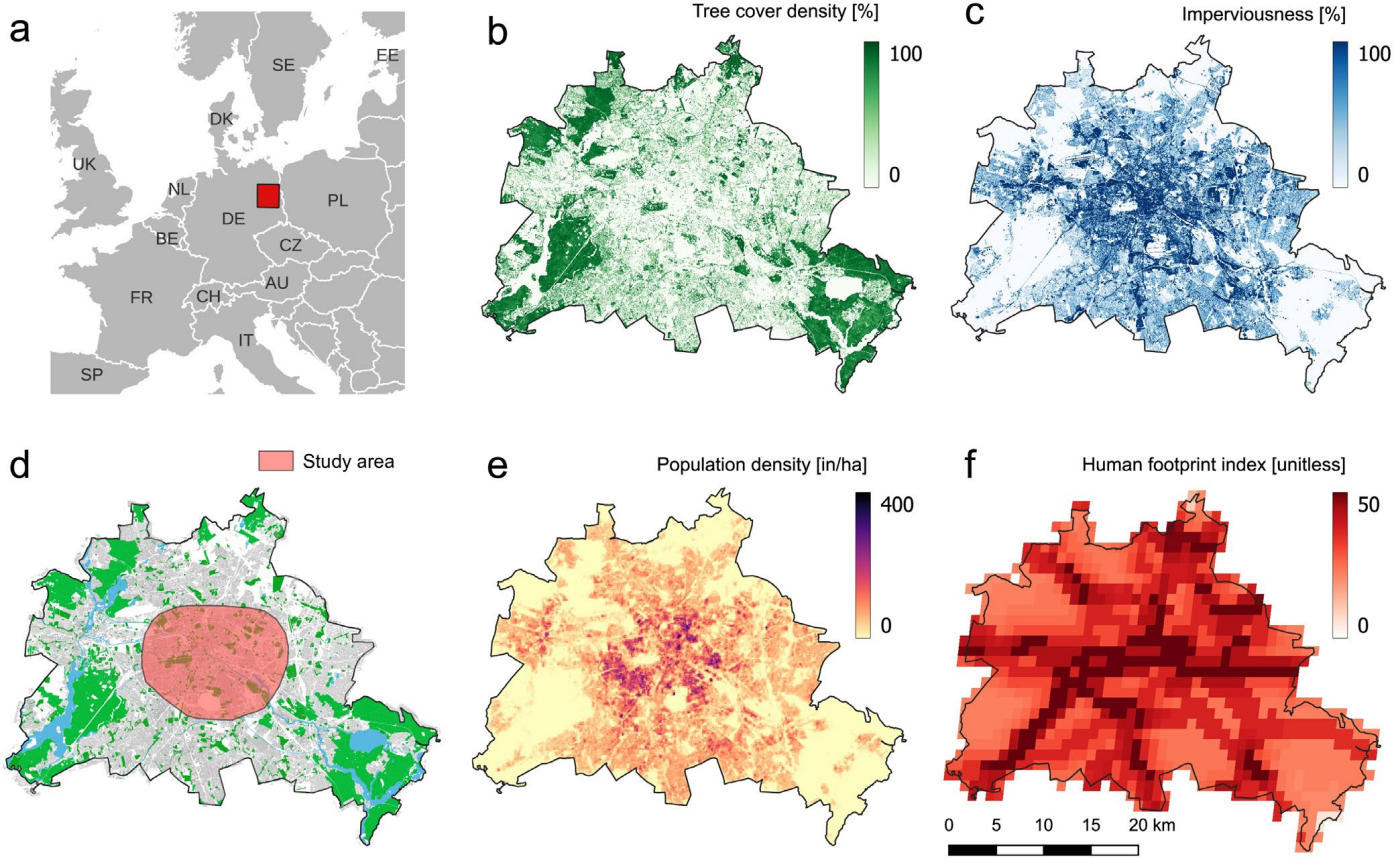
Study area and visualization of environmental predictors used to predict habitat suitability for breeding of urban Common Buzzards. (a) Situation of the study area (red square) in Germany (DE) in central Europe. (d) Study area (red circle, ca. 217 km^2^) with monitored breeding pairs covering the city centre of Berlin (ca. 890 km^2^). Buildings and residential areas are displayed in gray, green spaces with tree vegetation in green and water bodies in blue. (b, c, e, f) Geographical distribution of environmental predictors within the administrative boundaries of Berlin, including tree cover density [%] (b), imperviousness [%] (c), population density [in/ha] (e) and human footprint index [unitless] (f). All maps are north‐oriented.

In order to investigate the influence of the environment on the choice of breeding sites of urban Common Buzzards, we analyzed nest location data of 225 (20–30 per year) independent breeding events. Nest locations were systematically collected over seven consecutive breeding seasons between 2018 and 2024 in a 217 km^2^ study area covering the city centre of Berlin (Figure 2d). Potential breeding sites were systematically scanned for evidence of nest building and breeding activity each year between February and May. We only considered breeding pairs with confirmed evidence of nest building activity and for which both individuals were present within the nesting territory in April.

#### 2.3.2 Environmental Predictor Variables

In order to characterize the breeding habitat conditions, we used a total of six predictor variables of three classes, including (a) the habitat composition, (b) available habitat structures, and (c) human impact (Table 2). All predictors for these classes were rescaled to a spatial resolution of 50 m. In order to account for differences in the spatial scale at which predictors might affect breeding site selection, we aggregated predictor variables at three different spatial scales, with the nest coordinates serving as the focal point: firstly, at the local scale (50 m radius), which reflects small‐scaled habitat features for nesting; secondly, at the scale of the nesting territory (200 m radius), which reflects habitat properties within a broader range of nest sites; and thirdly, at the home‐range scale (1000 m radius), which reflects broad‐scaled habitat composition within the parental foraging area during breeding (Mateo‐Sánchez et al. 2013). We chose radii for the respective scales on the basis of reviewed home range and nesting territories sizes of Common Buzzards in Europe (Walls and Kenward 2020).

**TABLE 2:** Environmental variables used to predict habitat suitability for breeding of urban Common Buzzard breeding pairs.

Class	Predictor variable	Description	Spatial scale
Habitat composition	Habitat type (qualitative, 6 levels)	Residential, urban mixed, natural mixed, city parks, cemeteries, and others	—
	Imperviousness (quantitative)	Mean surface sealing [0%–100%]	Nesting territory, home range
Habitat structure	Tree cover density (quantitative)	Mean tree cover density [0%–100%]	Local, nesting territory
Human impact	Human footprint index (quantitative)	Global, dimensionless index of human pressure	Nesting territory, home range
	Population density (quantitative)	Mean human population density [inhabitants/ha]	Local, nesting territory, home range

*Note:* Spatial scales refer to the radius around the focal nest, at which values were aggregated: Local = 50 m; nesting territory = 200 m; home range = 1000 m.

For the class “habitat composition,” we classified the habitat type of the breeding site as an urban–rural gradient, encompassing six categories: residential areas, urban mixed areas, natural mixed areas, city parks, cemeteries and others (see Supporting Information S5 for details about generating predictors). We further obtained mean tree cover density (at both the local and nesting territory scale) and the mean imperviousness (at both the nesting territory and home range scale) from the Copernicus online database (https://land.copernicus.eu) in order to reflect the availability of natural vegetation for breeding and foraging (see Figure 2b,c).

We integrated the level of human impact by accessing data on human population density from the Copernicus online database (Figure 2e). We also incorporated the “Human Footprint Index”, which is a global, dimensionless index of human pressure on the land surface (see Figure 2f) that is calculated from eight different variables of human impact, including built environments, electric infrastructure, crop and pasture lands, roads and railways and others (Centre for International Earth Science Information Network [CIESIN], Venter et al. 2016).

We tested all predictors for collinearity and only retained the variable yielding superior model performance for variable pairs with Pearson's |*r*| > 0.7 (Dormann et al. 2013). Thus, we only retained the predictor variables habitat type, tree cover density (local scale), population density (local scale), human footprint (home‐range scale) and imperviousness (home‐range scale) for model building (see Supporting Information S6).

#### 2.3.3 Mapping Habitat Suitability for Breeding

In order to predict and map suitable breeding habitat, we ran a series of maximum entropy models (Maxent), a commonly used method used for species distribution models (Bluhm et al. 2023; Elith et al. 2011). We sampled a substantial number of background points (ratio of 10:1 to the number of known breeding sites) as random nest locations within the study area, with the exclusion of water area (Phillips et al. 2009).

We systematically tested different model parameterizations in relation to alternative correlating predictors, feature selection, and regularization multiplier. We considered the following feature combinations: (a) linear, quadratic, product, and hinge (LQPH), (b) hinge and product, as well as (c) solely hinge. We applied a vector of regularization multipliers (1,2,3,4,5) (Elith et al. 2010). All models were run with a maximum of 2500 iterations using the R package *dismo* (Hijmans et al. 2024). We evaluated model performance using the mean area under the curve (AUC) and validated all models via a 10‐fold internal cross‐validation by randomly sampling 10% of the breeding site and background data as test data in each fold (Pearce and Ferrier 2000).

Following model evaluation, we used the model with the best fit based on AUC (i.e., highest AUC) and True Skill Statistics (TSS = sensitivity + specificity ‐ 1) to predict continuous habitat suitability in both our study region and extrapolated across the entire city area. The continuous habitat suitability predictions were then converted into binary maps (suitable/non‐suitable) using the average threshold maximizing the sum of sensitivity and specificity (max_sss_, Liu et al. 2013; Scherrer et al. 2018). The resulting output served as a representation of potential breeding environments for Common Buzzards in the study region and across the entire city area. We excluded areas of forest located in the peripheral parts of the city area from our predictions, as they were not existent in the city centre and our predictions could thus not be considered transferable to these regions. To assess suitable breeding habitat within the study area that was not occupied by breeding Common Buzzard pairs during the study period, we used a buffer with a radius of 1000 m (ca. 3.1 km^2^) around each occupied nest to reflect circular territory boundaries of existing breeding pairs (Walls and Kenward 1998, 2020). The resulting area was then excluded from the binary prediction map.

## 3 Results

### 3.1 Foraging Ecology of Juveniles

#### 3.1.1 Core Foraging Ranges

**FIGURE 3:**
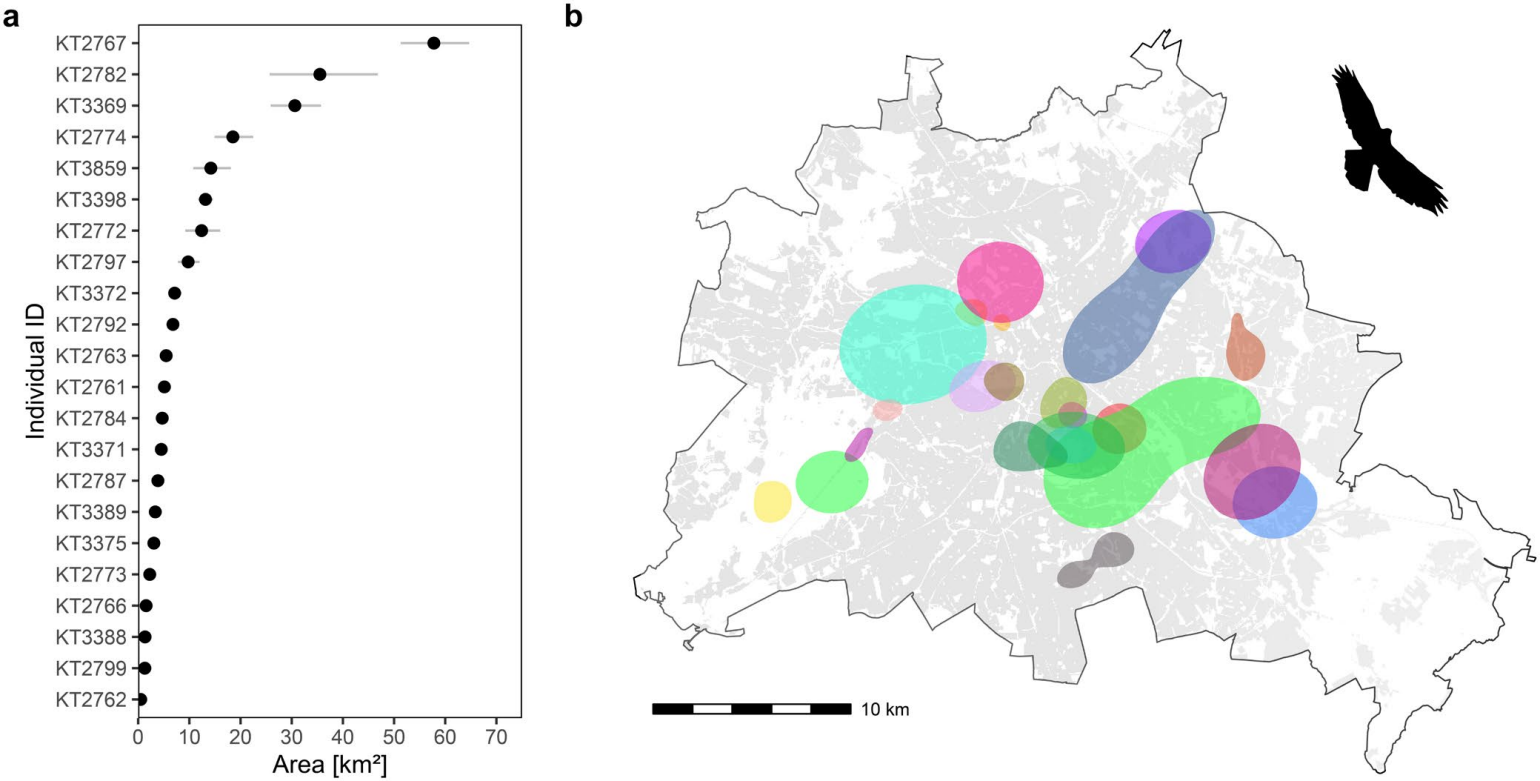
Estimates of wAKDEc 50% core foraging ranges (CFRs) for tracked juvenile Common Buzzards (*n* = 22) in the city of Berlin (Germany). (a) Ranked sizes of 50% CFR estimates [km^2^], with lines presenting the 95% CI. (b) Location of CFRs within the city region of Berlin. Gray areas (background) represent residential areas.

We estimated individual CFRs of 22 Common Buzzards based on wAKDEc during natal dispersal within the urban landscape (see Supporting Information S2 for model fits). The sizes of CFRs ranged between 0.5 and 57.8 km^2^, averaged an area of 11.0 km^2^ (±13.91 sd), and were mostly distributed across the centre of the city area (Figure 3). Particularly in this region, we observed strong overlap of core foraging areas between individuals, with some regions being occupied by up to three individuals simultaneously.

#### 3.1.2 Temporal Foraging Areas

Within the broader CFR across the city landscape, we characterized a total of 82 small‐scale TFAs occupied by Common Buzzards and further investigated the local conditions at these sites. More than half of TFAs were situated in either single courtyards or housing blocks in residential areas (35%, *n* = 29), or in recreational areas such as playgrounds and sports fields (12%, *n* = 10). A moderate proportion was also found in city parks (18%, *n* = 15; Table 3).

**TABLE 3:** Habitat‐specific sample sizes and spatio‐temporal properties of TFAs (*n* = 82). For each habitat type, group means, variation, and range are given with respect to the time span of use (95% quantile) and the area of TFAs.

Habitat type	No. of TFAs	Time span (days)		Area (ha)	
		Mean ± SD	Min–max	Mean ± SD	Min–max
City forest	7	261 ± 156	22–423	59 ± 16	40–86
High−/ railways	7	197 ± 151	18–404	24 ± 23	4–60
Allotments	4	337 ± 242	13–599	30 ± 37	3–83
Cemeteries	6	279 ± 279	21–689	8 ± 5	3–14
City parks	15	324 ± 242	30–662	32 ± 39	3–127
Depots	4	434 ± 227	119–607	7 ± 5	1–14
Recreation areas	10	284 ± 212	17–635	15 ± 23	2–77
Residential areas	29	259 ± 175	15–683	9 ± 7	1–34

*Note:* Habitat types are ordered by increasing level of human‐related disturbance (city forest = low; residential area = high).

The timespan over which TFAs were occupied averaged 274 days (±198), ranging from a few weeks to almost the entire natal dispersal phase of two years (Table 3). Concurrently, the area of TFAs averaged 20.4 ha (±26.1, or 0.2 ± 0.3 km^2^) and ranged between 1 ha and 127 ha. TFAs in densely populated regions, such as residential areas (9 ± 7 ha) and depots (7 ± 5 ha) exhibited the lowest area on average, often covering only a single courtyard approximately one hectare in size. Conversely, the utilized area of TFAs located in areas of low human population density, such as city forests, did not fall below 40 ha.

**FIGURE 4:**
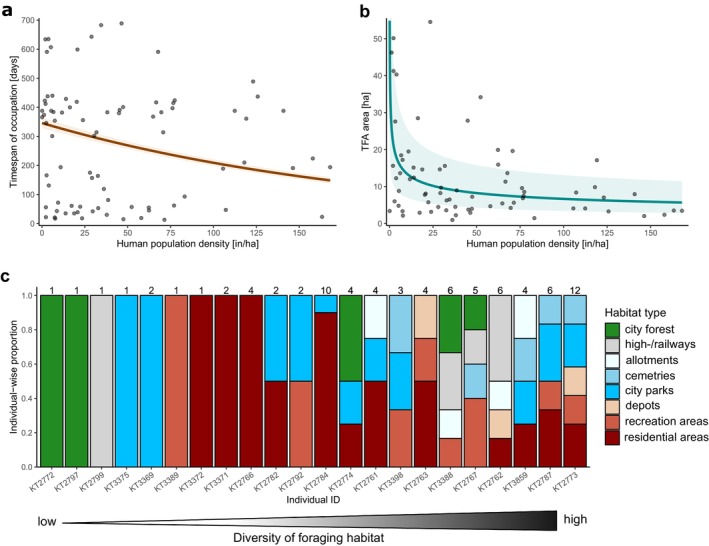
Metrics of temporal foraging areas (TFAs, *n* = 82) of 22 juvenile Common Buzzards established during natal dispersal in the city area of Berlin. (a) Relationship between the time span of occupation and average human population density of TFAs. Predictions are indicated by the dark orange line. Shaded areas indicate 67.45% confidence intervals (expected to contain 50% of values, that is the so‐called “probable error”) based on the prediction variance. Points mark individual TFAs of the underlying model data. See Supporting Information S3 for model estimates. (b) Relationship between the area and average human population density of TFAs (predictions and CI intervals as above). See Supporting Information S4 for model estimates. (c) Proportion of TFAs for each individual and all habitat types. Numbers on top of stacked bars indicate the total number of TFAs of the respective individual.

Along the gradient of human population density, we found a negative relationship with respect to the time span of occupation (LRT_PQL/L_ = 639.2, df = 1, *p* < 0.001; Figure 4a) as well as the utilized area of TFAs (LRT_PQL/L_ = 17.5, df = 1, *p* < 0.001; Figure 4b). TFAs in areas with high human population density (e.g., residential areas) were utilized over shorter time periods and were smaller in size compared to TFAs in less densely populated areas (e.g., city forest).

Common Buzzards also exhibited significant variability in terms of the number as well as diversity of habitat types of TFAs (Figure 4c). Approximately one third of Common Buzzards (27%, *n* = 6), established only single TFAs (i.e., low habitat diversity), which were primarily located in either urban forests or city parks. Conversely, 23% (*n* = 5) of individuals established five or more TFAs in at least four different types of habitats across the city landscape (i.e., high habitat diversity, Figure 4c).

#### 3.1.3 Composition of Temporal Foraging Areas

Subsequently, we assessed the environmental composition of established TFAs through in‐field observations. Along the gradient of human presence, we observed (a) a higher prevalence of features promoting rats, (b) more frequent direct observations of rats, as well as (c) increased levels of active pest control measures.

**FIGURE 5:**
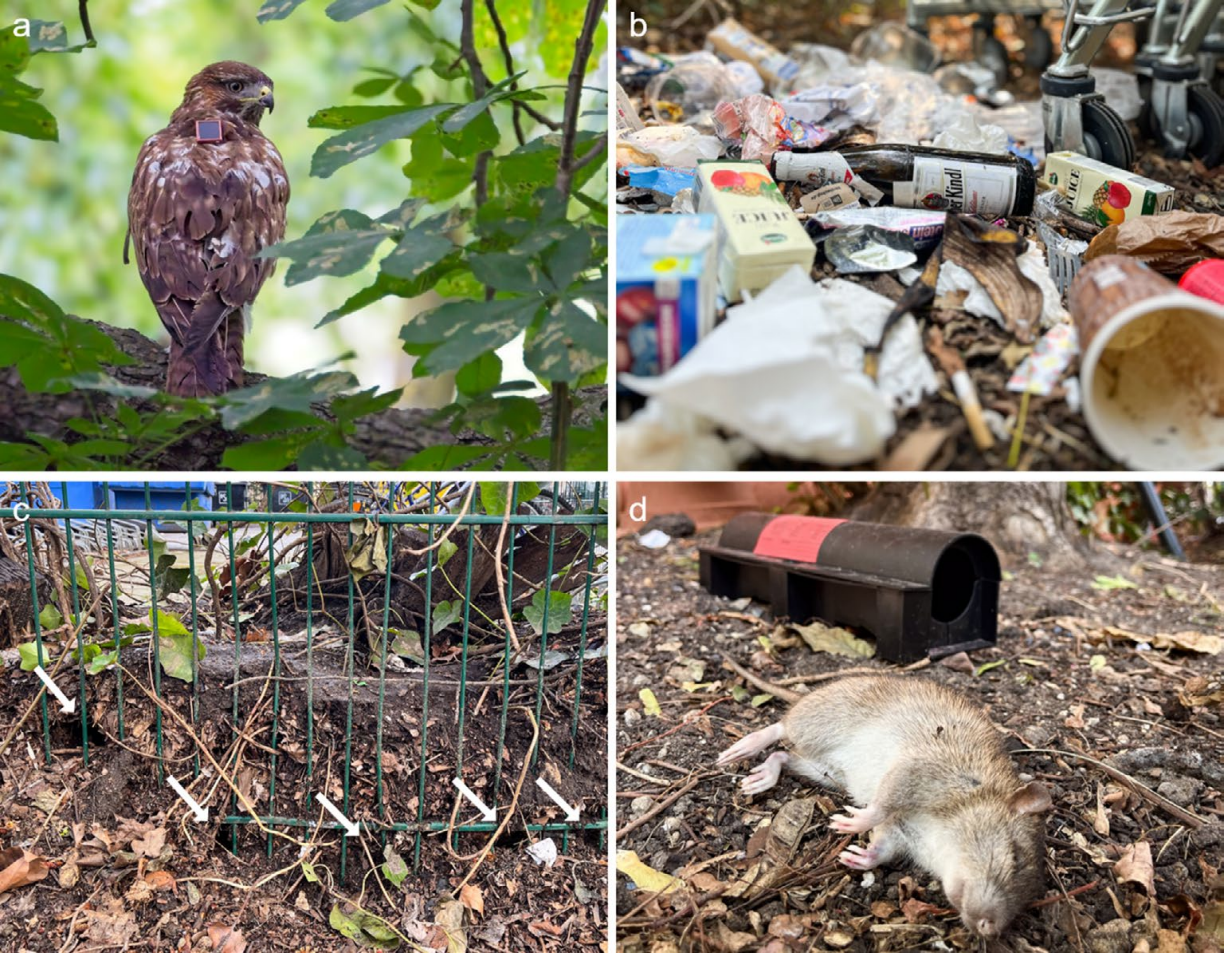
Exemplary environmental composition of temporal foraging areas (TFAs) utilized by Common Buzzards in Berlin (Germany), assessed through in‐field observations. (a) Juvenile Common Buzzard equipped with a GPS transmitter (backpack harness) perching on a branch in a courtyard of a residential area. Photograph by Stiftung Naturschutz Berlin/L. Kröner, used with permission. (b) Excessive waste left by humans outside of disposal bins in a residential district. (c) Burrows of synanthropic rats along a fence line in proximity to a bin storage area in a residential block. White arrows indicate visible entrances of burrows dug by rats. (d) Observation of a freshly deceased rat lying next to a baiting box that contains second‐generation anticoagulant rodenticides (SGARs) for pest control.

Particularly in areas with high levels of human presence, such as recreational and residential areas, almost all TFAs (95%, *n* = 37) either had rats detected during on‐site visits or at least displayed rat‐promoting features, including excessive bird feeding, public waste disposal, or nearby food sales (Figure 5). In city parks, almost all TFAs (93%, *n* = 14) exhibited features conducive to the presence of synanthropic rats.

**FIGURE 6:**
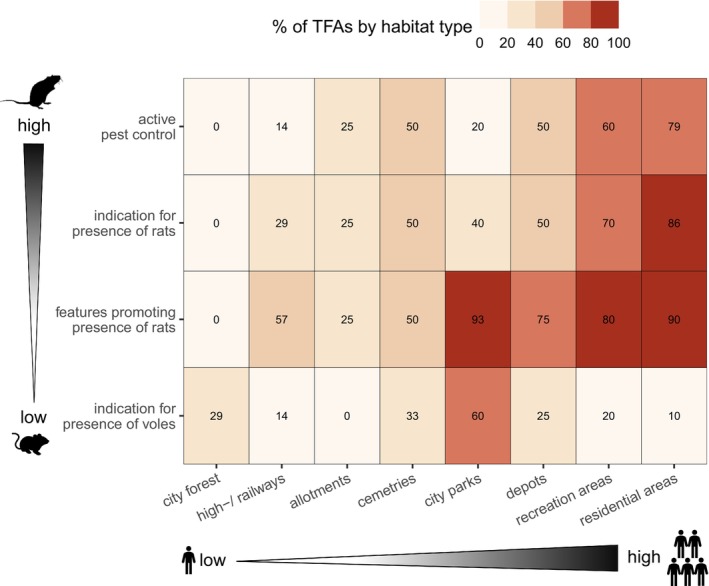
Environmental composition of temporal foraging areas (TFAs, *n* = 82) of 22 juvenile Common Buzzards established during natal dispersal in the city area of Berlin, assessed through in‐field observations. TFAs were categorized into eight habitat types and ranked according to the observed level of human presence (x‐axis). Numbers and associated colors of panels indicate the habitat type‐specific percentage of TFAs at which different environmental features regarding prey availability (y‐axis) were observed. Environmental features were ranked from indication of natural prey (e.g., voles) to high levels of human‐promoted prey, including active pest control measures. For example, in 93% of all TFAs located in city parks, we observed features promoting the presence of rats (see Table 1 for details).

Active pest control measures were observed at approximately half of all TFAs (48%, *n* = 39), and were most prominent in residential (79%, *n* = 23) and recreational areas (60%, *n* = 9), indicating high levels of rat abundance. Lowest levels of pest control measures were observed in city parks, along urban infrastructure, and in city forests. At cemeteries, half of the TFAs (*n* = 3) indicated the presence of rats in addition to active pest control measures. Concurrently, indications of the presence of natural prey, such as voles, were among the most prevalent (60%, *n* = 9) in city parks, with minimal levels observed across TFAs in all other habitat types (Figure 6).

### 3.2 Breeding Ecology of Adults

#### 3.2.1 Distribution of Breeding Sites

Based on a total of 225 breeding sites in the central area of Berlin, breeding pairs predominantly built their nest in city parks (41%, n_nest sites_ = 93), cemeteries (31%, n_nest sites_ = 69), and residential areas (15%, n_nest sites_ = 34). Other types of breeding habitats, including natural mixed areas with dominant shrub and tree vegetation and urban mixed areas with moderate anthropogenic use, accounted for 13% (n_nest sites_ = 29) of all records (see Supporting Information S7).

#### 3.2.2 Environmental Habitat Suitability for Breeding

Based on the best‐performing Maxent model, suitability of breeding habitat was primarily shaped by tree cover density at the local scale (62.8% model contribution) and the habitat type (31.1% model contribution), with environmentally suitable habitat generally associated with high tree cover density and habitats, such as city parks and cemeteries (see Supporting Information S8 and S9). All other predictors accounted for 6.1% of the total model contribution.

**FIGURE 7:**
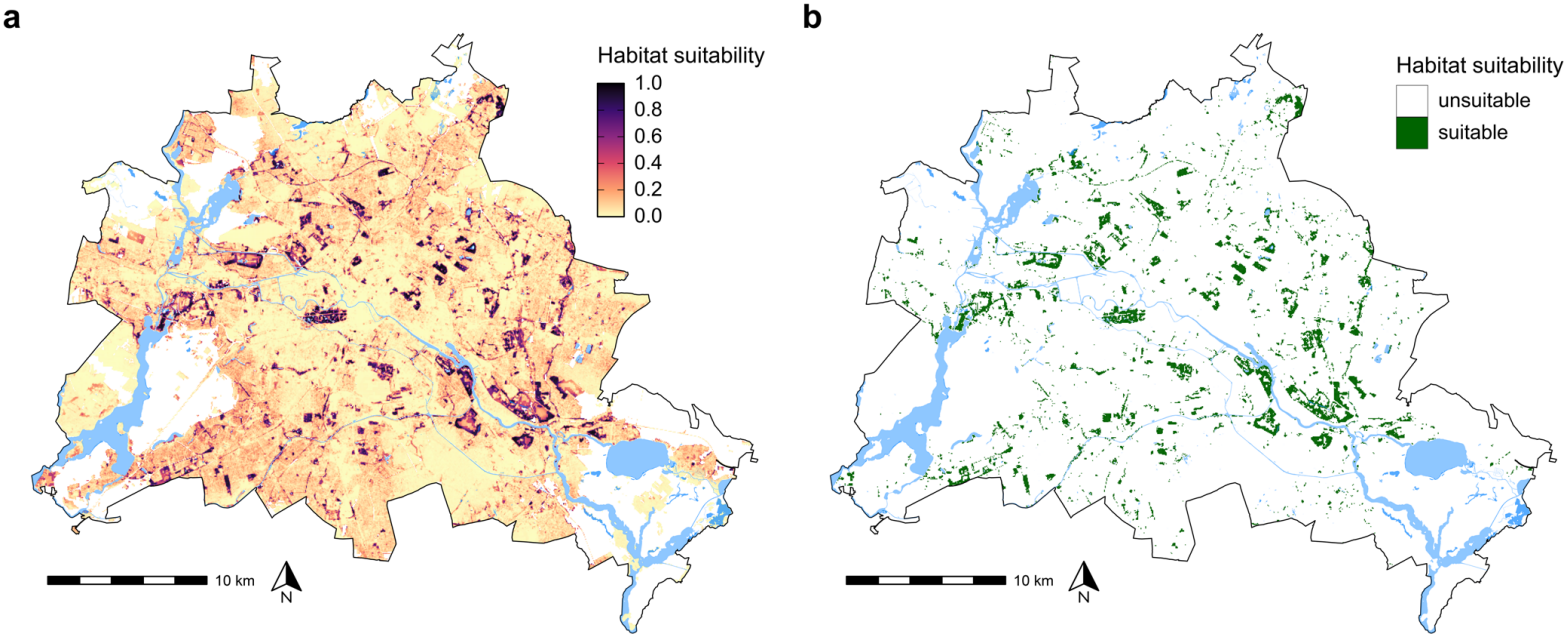
Potential environmentally suitable breeding habitat for Common Buzzards in the city area of Berlin (Germany). (a) Continuous output of the habitat suitability model across the city area (890 km^2^). The study area (ca. 217 km^2^) is located in the city centre, but is not displayed for better visibility of the model predictions (see Figure 2d). Habitat suitability was not mapped for core forests and pastureland (white patches) as these were non‐existent within the study area. (b) Binary output of the habitat suitability model. Water bodies are shown in blue for reference.

Within the study area (ca. 217 km^2^), the model predicted a total area of 17.3 km^2^ of suitable breeding habitat (8% of study area; Figure 7b), which mainly consisted of city parks (11.2 km^2^, 65%) and cemeteries (2.8 km^2^, 16%; see Supporting Information S10). Out of the 17.3 km^2^, 3.87 km^2^ (22%) were not occupied by territorial breeding pairs during the study period and thus remained as unoccupied suitable breeding habitat. Combined, city parks (1.7 km^2^, 45%), cemeteries (0.9 km^2^, 24%), and natural mixed habitats (0.8 km^2^, 21%) accounted for almost 90% of the suitable and unoccupied area (see S10).

Projected across the entire city area (ca. 890 km^2^), we mapped a total area of 45.41 km^2^ of suitable breeding habitat (5% of the city area), with city parks accounting for almost two‐thirds of the area (29.8 km^2^, 66%). Combined, cemeteries (4.6 km^2^, 10%), mixed natural habitats (5.5 km^2^, 12%), and mixed urban habitats (3.7 km^2^, 8%) accounted for approximately 30% of the total breeding habitat. The potential breeding habitat was characterized by significant fragmentation into smaller isolated patches. The patch size averaged 3 ha (±11) and ranged from 0.25 ha up to 1.91 km^2^.

## 4 Discussion

In the present study, we analyzed GPS tracking data and nest‐monitoring data of an urban top predator in a European metropolis. Our objectives were to assess the spatio‐temporal properties of both foraging areas for dispersing juveniles and breeding habitats of adult breeding pairs in order to understand how raptors utilize urban city landscapes. We discuss our results on foraging patterns and habitat suitability, before drawing conclusions about the implications for future management and conservation efforts.

### 4.1 Foraging Ecology of Juveniles

#### 4.1.1 Core Foraging Ranges

We found that CFRs utilized by juvenile Common Buzzards during natal dispersal were distributed across the city area and characterized by large variability in terms of the total area, as well as moderate degree of overlapping between multiple individuals. In rural landscapes, close proximity to, and even spatial overlap between, dispersing juveniles is commonplace, especially when resources are abundant (Arraut et al. 2021; Walls and Kenward 2020). In most raptor species, foraging sites are often utilized by multiple individuals, particularly during early life‐history phases such as natal dispersal (Arraut et al. 2021), during periods with severe environmental conditions (Passarotto et al. 2022), and for populations in strongly fragmented habitats (Martin et al. 2006). Our results indicate that in urban environments, the heterogeneous landscape and patchy distribution of foraging sites particularly promote the shared or sequential use of foraging sites by opportunistic predators like Common Buzzards, allowing several individuals to exploit abundant resources simultaneously.

#### 4.1.2 Temporal Foraging Areas

Our analyses revealed distinct patterns regarding the temporal and spatial extent of foraging areas in urban environments: we found that most TFAs were located in residential or recreational areas and often comprised only single courtyards with an area of only one hectare. TFAs in such densely populated areas were generally occupied over shorter timespans and were substantially smaller in area than TFAs in more natural habitats. Although juveniles often display movements on larger spatial scales compared to adult individuals (Hemery et al. 2024; Kocina and Aagaard 2021), we propose that the reduced movement extent in residential areas may be indicative of particularly profitable foraging conditions and/or high prey abundance. These profitable foraging conditions may comprise a combination of reduced necessity for changes of location (Fargallo et al. 2020) and/or increased foraging success, for example, due to increased hunting efficiency (Rutz 2006). Thus, even single courtyards or isolated depots may allow opportunistic raptors to exploit abundant, yet temporarily limited prey availability within a confined area, obviating the requirement for displacement to a different location (Orgeret et al. 2024). Contrarily, more natural foraging habitat may be exploited over longer time periods, but at the expense of higher movement costs.

Concurrently, we found high variation in the quantity and habitat diversity of TFAs among individuals. In general, local prey availability (Mirski et al. 2024) as well as increased foraging success (Mak et al. 2021; Planillo et al. 2015) may promote fidelity to a foraging site. Conversely, perceived human disturbance (Mak et al. 2023) and intra‐specific competition for high‐quality foraging sites can reduce site fidelity and force less competitive individuals to move to alternative locations (Schindler et al. 2012; Walls and Kenward 2020). The observed TFA patterns may thus reflect the spectrum of competitiveness and fitness among dispersing juveniles (Ferrer and Morandini 2017). For instance, particularly competitive individuals may occupy a single high‐quality foraging site, while weaker juveniles who are unable to occupy such locations are forced to explore wider areas and visit a larger number of low‐quality TFAs at the expense of increased energetic cost (Rivera et al. 2022). Such behavior would also correlate with a larger diversity of habitat types, through the opportunistic behavior of juveniles searching for lucrative foraging sites.

#### 4.1.3 Composition of Temporal Foraging Areas

In a more detailed analysis, we characterized the site‐specific composition of TFAs through in‐field observations. We found the highest prevalence for synanthropic rats or features promoting them in TFAs that were located in residential and recreational areas. Human refuse (e.g., food waste, excessive bird feeding, etc.) has been shown to be the primary cause for attracting synanthropic rats around the globe and is often the result of high human population density and insufficient waste control management (Shukla and Wilmers 2024). Since rats generally constitute a highly profitable prey for both rodent specialist and generalist predators (Torre et al. 2024; Walls and Kenward 2020), their abundance likely constitutes one of the main attracting factors causing Common Buzzards to occupy foraging sites in the immediate vicinity to humans (Boal and Dykstra 2018; Hindmarch and Elliott 2015). Complementary, sit‐and‐wait predators may take advantage of buildings or anthropogenic structures as vantage points and perches, thus additionally increasing their hunting success (Mak et al. 2023). In this context, we underline that our assessment of prey availability, based on direct field observations, was restricted to qualitative indicators (presence/absence) of prey rather than quantitative estimates of population density or relative abundance. Thus, any inference regarding fine‐scale variation in prey availability among TFAs is limited in the context of this study, and we were not able to investigate any responses of the tracked juveniles to varying local prey densities. Consequently, our findings should be interpreted as evidence for associations between urban habitat features and the occurrence of potential prey for Common Buzzards, rather than as precise measures of prey availability.

Our results also suggest that city parks may still represent rather natural foraging habitats and do not appear to accommodate rat populations at scales comparable to residential areas in the context of this study. Yet, the excessive food waste found in almost all TFAs in city parks, as a likely result of their intense use as retreat and recreational spaces by humans, suggests that even city parks offer the nutritional and structural resources rats rely on (Richardson et al. 2025; Shukla and Wilmers 2024). The small‐scale mosaic of green urban spaces in the urban residential matrix may thus promote the progressive spread of rats into more natural urban areas and nudge urban predators to adapt their movement patterns, foraging behavior, and diet composition (Rooney and Montgomery 2013; Torre et al. 2024).

Linked to this, our findings further revealed that, at most TFAs juveniles foraged at in the city area, active pest control measures in the form of baiting boxes were present. In the context of this study, it remains unknown whether the high prevalence of evident rodent management, particularly in depots, recreational, and residential areas is linked to an elevated risk for exposure to secondary rodenticide intoxication, that is, through the consumption of poisoned rats in residential areas by predators (Elliott et al. 2022). Although high levels of urbanization have been identified as a reliable predictor of AR exposure on a broader scale of habitat composition for raptor species (Badry et al. 2021; López‐Perea et al. 2015, 2019), it is possible that this causal relationship may gradually dissolve at the level of small‐scale, heterogeneous environments in cities. For instance, urban rat populations may even propagate into more natural habitat in proximity to residential areas, thus increasing the risk for secondary AR poisoning even in these rather natural areas (Badry et al. 2021; Hughes et al. 2013). We propose that future research should investigate to what extent urban raptor populations are exposed to lethal or sub‐lethal concentrations of ARs, and which consequences for health and mortality emerge for urban predators (Badry et al. 2020; López‐Perea and Mateo 2018).

While we analyzed the foraging behavior of juvenile individuals during natal dispersal, the findings and conclusions are likely also representative at the population level, that is, they can be applied across age groups (Hemery et al. 2024). Given their highly opportunistic foraging behavior, we hypothesize that adult individuals also exploit high‐quality foraging sites with similar environmental conditions in order to decrease energetic costs (Planillo et al. 2015; Schindler et al. 2012). This is particularly true of breeding pairs of central‐place foragers during the chick‐rearing period (Engler et al. 2021; Stephens and Krebs 1986). Although Common Buzzards typically display strong territorial behavior (Schindler et al. 2012; Walls and Kenward 2020), the widespread availability of foraging patches in urban landscapes may enable both juvenile and adult individuals to exploit local profitable resources simultaneously, thus potentially reducing intra‐specific competition across age classes (Kettel et al. 2018).

### 4.2 Breeding Ecology of Adults

#### 4.2.1 Environmental Habitat Suitability for Breeding

By modeling the suitability of breeding habitat for urban Common Buzzards, we aimed to understand the environmental properties of nest locations and to predict suitable breeding habitat within urban areas. Our model predictions suggest that even single courtyards with few mature trees, located in residential areas and in proximity to humans, can represent suitable breeding habitat for urban Common Buzzards. This is supported by the small, yet noticeable proportion of nest sites that were located in single courtyards in this study. Urban breeding sites are often considered as alternative low‐quality habitat, particularly when populations reach breeding densities and high‐quality habitats are already occupied (Newton 1979). Thus, urban breeding sites do not necessarily correlate with high habitat quality and may even represent ecological traps (Sumasgutner et al. 2014). Yet, our results indicate that even residential areas can represent suitable, although extreme, breeding sites for raptors, which may be occupied by particularly bold individuals that are more tolerant of human disturbance (Boal and Dykstra 2018; Møller et al. 2012). Raptors breeding in residential areas may benefit from an increased foraging efficiency and/or reduced energetic expenditure due to shorter foraging trips and hunting in areas with higher prey abundance (Leveau et al. 2022; Merling de Chapa et al. 2020). Similar choices of breeding sites have been documented for Northern Goshawks (Merling de Chapa et al. 2020; Rutz 2004; Solonen 2008), Cooper's Hawks (Cooper et al. 2021; Millsap 2017), and Red‐tailed Hawks ( *Buteo jamaicensis* , Stout et al. 2006). Considering that nest locations in residential areas are often inaccessible and more difficult to monitor, it is likely that the here‐reported proportion of breeding locations in residential areas is generally underestimated.

Most nests in the present study were located in city parks and cemeteries, accounting for 72% of breeding sites. This is consistent with previous research identifying city parks and cemeteries as favorable breeding grounds for raptor species in urban environments (Boal and Dykstra 2018; McPherson and Sumasgutner 2021). In such areas, raptors benefit from mature tree stands for building nests and lower human disturbance when compared to nearby residential or industrial areas (Mirski 2020; Natsukawa et al. 2017). Urban green spaces thus provide essential habitat supporting raptor reproduction. Their loss as a result of the land transformation to meet human needs may have negative effects of an as‐yet‐unpredictable extent. Since urban green spaces support a wide range of ecosystem services across various taxa, their conservation will not only support urban raptor populations but also ultimately assist in fostering biodiversity in general (Gallo and Fidino 2018; Wang and Xu 2024). Even from a human standpoint, they are vital for establishing microclimates, serving as recreational retreats, and implementing the societal demands for urban development in cities (Martens et al. 2022; Zhang et al. 2024). The future demographic response of urban raptor populations, at least in part, may depend on the preservation of urban green spaces, both in and around residential areas, as well as on the behavioral plasticity to forage and breed in proximity to humans. More specifically, it depends on whether the advantages of resource availability will be outweighed by the tolerance limits for human‐related disturbance (Boal and Dykstra 2018; Kark et al. 2007).

Our predictions further indicate that around a quarter of the suitable habitat within the study area is currently not occupied by breeding pairs. We therefore conclude that the population density may not yet have reached a population limit and may increase further over time. The future occupation of these potential breeding sites thus seems likely but depends on the demographic response to future habitat changes (Arraut et al. 2021; Björklund et al. 2015), interspecific competition (Björklund et al. 2016), and resource availability (Gryz and Krauze‐Gryz 2021).

## 5 Conclusion

In the present study, we aimed to understand how raptors utilize the urban city landscape for foraging and breeding. Our results show that Common Buzzards display contrasting habitat use between foraging and breeding in urban environments. Foraging areas were mostly located in residential areas with high levels of human activity and a high abundance of rats. Raptors may benefit from such foraging areas because they offer high prey abundance in confined spaces, enabling them to increase their foraging efficiency and reduce their daily energy expenditures by employing a sit‐and‐wait strategy. Individuals capable of tolerating the associated perceived human disturbance at these sites can exploit the advantages compared to more natural foraging areas. This applies to both dispersing individuals, for whom foraging success—and thus survival—is key during the early phases of their life history, and to breeding pairs with high demand for prey intake for chick‐rearing during the breeding season.

Concurrently, the present study contributes to the available evidence that urban areas can provide suitable breeding habitats for raptors. Even residential areas can represent almost extreme habitats for disturbance‐tolerant raptors, where they benefit from the proximity to high‐quality foraging grounds. The conservation of mature tree stands, both in city parks and residential areas, is thus of paramount importance for the preservation of raptor species, which play a pivotal role in urban ecosystems.

In conclusion, the term “living on the edge” may not only refer to the selection of edge structures during foraging in cities; in a figurative sense, it also summarizes the fine line between opportunities and risks that opportunistic predators face in urban environments. As a foundation for the protection and conservation of urban wildlife, we emphasize the need for studies investigating the population‐level impacts of pest control measures on urban predators through poisoning along the food chains, as well as the role of urban green spaces in breeding and reproduction.

## Author Contributions

**Marc Engler:** conceptualization (equal), data curation (lead), formal analysis (lead), funding acquisition (lead), investigation (lead), methodology (equal), project administration (equal), resources (lead), software (lead), validation (equal), visualization (lead), writing – original draft (lead), writing – review and editing (equal). **Oliver Krone:** conceptualization (equal), investigation (supporting), methodology (equal), project administration (equal), supervision (lead), validation (equal), writing – original draft (supporting), writing – review and editing (equal).

## Funding

The study was funded by private donations made to the Wild Bird Rehabilitation Center of the NABU Landesverband Berlin e.V.

## Conflicts of Interest

The authors declare no conflicts of interest.

## Supporting information


**Data S1:** ece373097‐sup‐0001‐Supinfo.docx.

## Data Availability

The data and R code that support the findings of this study are available on GitHub: https://github.com/marcengler/Engler_Krone_2026_Ecology_and_Evolution_review.
