# Supplementary material for: Living on the Edge: Juvenile Foraging and Adult Breeding Ecology of an Aerial Top Predator in Urban Environments
Source: Ecol Evol. 2026 Feb 11;16(2):e73097. doi: 10.1002/ece3.73097 (PMC12893809; doi:10.1002/ece3.73097)

# Supplementary material

**Living on the edge: juvenile foraging and adult breeding ecology of an aerial top predator in urban environments**

**S1: Individual-level overview of the dataset used to analyse core foraging ranges (CFRs) and temporal foraging areas (TFAs) of Common Buzzards (n = 22) in urban environments.** The listed data sets include all GPS positions after data processing (see 2.2 for details).

| Individual ID | sex | Total fixes | Tracking days | Tracking seasons |
| --- | --- | --- | --- | --- |
| KT2761 | m | 11853 | 421 | 2 |
| KT2762 | m | 10785 | 378 | 1 |
| KT2763 | f | 6311 | 362 | 2 |
| KT2766 | f | 2475 | 136 | 1 |
| KT2767 | f | 5025 | 267 | 1 |
| KT2772 | m | 1811 | 76 | 2 |
| KT2773 | m | 12675 | 447 | 2 |
| KT2774 | m | 12760 | 331 | 2 |
| KT2782 | m | 2736 | 55 | 1 |
| KT2784 | f | 7221 | 353 | 1 |
| KT2787 | m | 9162 | 378 | 1 |
| KT2792 | f | 3160 | 343 | 2 |
| KT2797 | f | 4271 | 196 | 1 |
| KT2799 | m | 1055 | 21 | 1 |
| KT3369 | m | 3212 | 184 | 1 |
| KT3371 | f | 1523 | 84 | 1 |
| KT3372 | f | 1451 | 67 | 1 |
| KT3375 | m | 3020 | 174 | 1 |
| KT3388 | m | 10024 | 466 | 2 |
| KT3389 | f | 4996 | 358 | 2 |
| KT3398 | f | 3445 | 211 | 1 |
| KT3859 | f | 1460 | 69 | 1 |

# S2: Model results from fitted aAKDEc estimating 50% core foraging ranges of Common Buzzards. Abbreviations for movement models are as follows: OU = Ornstein-Uhlenbeck, OUF = Ornstein-Uhlenbeck (foraging process), IID = independently and identically distributed. dRMSPE = the root mean squared prediction error. DOF area = the effective sample size.

| Animal ID | Movement model | dAICc | dRMSPE [m] | DOF area |
| --- | --- | --- | --- | --- |
| KT2761 | OUFanisotropic | 0 | 727,5 | 596,7 |
| KT2761 | OUanisotropic | 730,8 | 790,3 | 473,2 |
| KT2761 | OUF | 832,8 | 749,1 | 615,8 |
| KT2761 | OUfanisotropic | 7295,8 | 0 | 3313,8 |
| KT2762 | OUFanisotropic | 0 | 153 | 1629,9 |
| KT2762 | OUF | 68 | 161,9 | 1638,6 |
| KT2762 | OUanisotropic | 783,5 | 156 | 1341 |
| KT2762 | OUfanisotropic | 2284,6 | 0 | 3895,5 |
| KT2763 | OUFanisotropic | 0 | 265,3 | 884,2 |
| KT2763 | OUF | 24,3 | 239 | 899,5 |
| KT2763 | OUanisotropic | 356,6 | 233,1 | 763,1 |
| KT2763 | OUfanisotropic | 3375,3 | 0 | 2486,7 |
| KT2766 | OUFanisotropic | 0 | 54,7 | 584,7 |
| KT2766 | OUF | 29,7 | 72,2 | 588,3 |
| KT2766 | OUanisotropic | 152,6 | 12,6 | 521,5 |
| KT2766 | OUfanisotropic | 687,1 | 0 | 1017,3 |
| KT2767 | OUFanisotropic | 0 | 976,5 | 286,4 |
| KT2767 | OUF | 76 | 1279 | 275,3 |
| KT2767 | OUanisotropic | 131,5 | 914,8 | 255,8 |
| KT2767 | OUfanisotropic | 5572,3 | 0 | 1391,2 |
| KT2772 | OUanisotropic | 0 | 1,6 | 50,6 |
| KT2772 | OUFanisotropic | 2 | 1,6 | 50,3 |
| KT2772 | OU | 40 | 2,1 | 41,5 |
| KT2772 | OUF | 41,4 | 2 | 42,9 |
| KT2772 | OUfanisotropic | 823 | 0 | 460,2 |
| KT2772 | IIDanisotropic | 6030 | 0,4 | 1810 |
| KT2773 | OUFanisotropic | 0 | 124,2 | 2429 |
| KT2773 | OUF | 145,1 | 113,5 | 2468,1 |
| KT2773 | OUfanisotropic | 1634 | 0 | 4430,4 |
| KT2773 | OUanisotropic | 2411,6 | 132,3 | 1656,8 |
| KT2774 | OUFanisotropic | 0 | 2,1 | 92,3 |
| KT2774 | OUF | 293,4 | 2 | 95,7 |
| KT2774 | OUanisotropic | 1015,8 | 2,4 | 66,6 |
| KT2774 | OUfanisotropic | 12488,9 | 0 | 2284 |
| KT2782 | OUFanisotropic | 0 | 2,3 | 43,4 |
| KT2782 | OUF | 121,7 | 2,3 | 43,4 |
| KT2782 | OUanisotropic | 338,3 | 2,5 | 28,9 |
| KT2782 | OUfanisotropic | 1527,2 | 0 | 472,4 |
| KT2784 | OUFanisotropic | 0 | 860,5 | 455,7 |
| KT2784 | OUanisotropic | 194,3 | 886,7 | 418,4 |
| KT2784 | OUF | 339,3 | 970,4 | 443,5 |
| KT2784 | OUfanisotropic | 5610,8 | 0 | 2655,5 |
| KT2787 | OUFanisotropic | 0 | 565,9 | 723 |
| KT2787 | OUanisotropic | 363,8 | 565,2 | 623,1 |
| KT2787 | OUF | 579,2 | 779,6 | 681,1 |
| KT2787 | OUfanisotropic | 5789,5 | 0 | 2995,1 |
| KT2792 | OUanisotropic | 0 | 375,8 | 538,3 |
| KT2792 | OUFanisotropic | 20,9 | 787,6 | 245,8 |
| KT2792 | OU | 216,8 | 500,9 | 519,7 |
| KT2792 | OUF | 227,8 | 787,6 | 314,9 |
| KT2792 | OUfanisotropic | 3152,3 | 0 | 1765,5 |
| KT2792 | IIDanisotropic | 12932,9 | 985,1 | 3159 |
| KT2797 | OUFanisotropic | 0 | 1,6 | 81,7 |
| KT2797 | OUanisotropic | 104 | 1,7 | 67,9 |
| KT2797 | OUF | 209,7 | 1,4 | 84,2 |
| KT2797 | OUfanisotropic | 2610,1 | 0 | 910,9 |
| KT2799 | OUFanisotropic | 0 | 126,3 | 152,2 |
| KT2799 | OUanisotropic | 35,5 | 122,6 | 124,6 |
| KT2799 | OUF | 121,5 | 90,2 | 157,6 |
| KT2799 | OUfanisotropic | 257,5 | 0 | 359,4 |
| KT3369 | OUFanisotropic | 0 | 888,1 | 149,2 |
| KT3369 | OUanisotropic | 80,5 | 851,1 | 127,4 |
| KT3369 | OUF | 589,6 | 1418 | 142,9 |
| KT3369 | OUfanisotropic | 2346 | 0 | 753,4 |
| KT3371 | OUanisotropic | 0 | 492,7 | 241,1 |
| KT3371 | OUFanisotropic | 0,3 | 487,7 | 249,3 |
| KT3371 | OU | 132,9 | 684,3 | 226 |
| KT3371 | OUF | 134,9 | 674,4 | 231,3 |
| KT3371 | OUfanisotropic | 408,1 | 226,1 | 608,4 |
| KT3371 | IIDanisotropic | 3076,6 | 0 | 1522 |
| KT3372 | OUanisotropic | 0 | 454,6 | 134 |
| KT3372 | OUFanisotropic | 2 | 435 | 140,4 |
| KT3372 | OU | 29,3 | 419,9 | 138,4 |
| KT3372 | OUF | 31,2 | 401,2 | 144,8 |
| KT3372 | OUfanisotropic | 880,4 | 0 | 546,5 |
| KT3372 | IIDanisotropic | 5652 | 199,8 | 1450 |
| KT3375 | OUFanisotropic | 0 | 157,7 | 559,8 |
| KT3375 | OUanisotropic | 6,9 | 156,8 | 530,1 |
| KT3375 | OUF | 87,3 | 196,7 | 553,9 |
| KT3375 | OUfanisotropic | 1042,4 | 0 | 1300,4 |
| KT3388 | OUFanisotropic | 0 | 101,5 | 2374,4 |
| KT3388 | OUanisotropic | 91,2 | 100,1 | 2206,3 |
| KT3388 | OUF | 213,8 | 106,5 | 2363,6 |
| KT3388 | OUfanisotropic | 3598,3 | 0 | 5203,2 |
| KT3389 | OUFanisotropic | 0 | 263,8 | 726,7 |
| KT3389 | OUanisotropic | 232,3 | 266,4 | 650,4 |
| KT3389 | OUF | 364,8 | 213,4 | 776 |
| KT3389 | OUfanisotropic | 3543,8 | 0 | 2224,1 |
| KT3398 | OUFanisotropic | 0 | 612,7 | 310,8 |
| KT3398 | OUanisotropic | 90,4 | 572,9 | 273,2 |
| KT3398 | OUF | 478,9 | 1155,5 | 286 |
| KT3398 | OUfanisotropic | 2092,3 | 0 | 1033,3 |
| KT3859 | OUFanisotropic | 0 | 1,5 | 57,3 |
| KT3859 | OUF | 13,4 | 1,4 | 58 |
| KT3859 | OUanisotropic | 126,2 | 1,6 | 42,4 |
| KT3859 | OUfanisotropic | 613,4 | 0 | 328,2 |
| KT2761 | OUFanisotropic | 0 | 727,5 | 596,7 |
| KT2761 | OUanisotropic | 730,8 | 790,3 | 473,2 |
| KT2761 | OUF | 832,8 | 749,1 | 615,8 |
| KT2761 | OUfanisotropic | 7295,8 | 0 | 3313,8 |

**S3:** **Model parameter estimates for the GLMM modelling the timespan of occupation (days) of temporal foraging areas (TFAs) of Common Buzzards in urban environments.** The model was fitted on a dataset of 82 TFAs from 22 individuals, located in the city of Berlin (Germany). The intercept corresponds to the value of ‘0’ for the mean human population density. Standard Errors for parameter estimates are displayed as conditional estimates.

| Fixed effects |  |  |
| --- | --- | --- |
| Term | Estimate | Cond. SE |
| Intercept | 5.011 | 0.229 |
| Human population density | -0.005 | 0.002 |
| Random effects |  |  |
| Term |  | Variance (λ) |
| Individual ID |  | 1.141 |

**S4:** **Model parameter estimates for the GLMM modelling the utilized area (ha) of temporal foraging areas (TFAs) of Common Buzzards in urban environments.** The model was fitted on a dataset of 82 TFAs from 22 individuals, located in the city of Berlin (Germany). The intercept corresponds to the value of ‘0’ for the mean human population density. Standard Errors for parameter estimates are displayed as conditional estimates.

| Fixed effects |  |  |
| --- | --- | --- |
| Term | Estimate | Cond. SE |
| Intercept | 3.505 | 0.259 |
| Human population density | -0.306 | 0.069 |
| Random effects |  |  |
| Term |  | Variance (λ) |
| Individual ID |  | 0.396 |

# S5: Generation of predictor variables used in the habitat suitability models.

We accounted for variation in the spatial scale at which different variables influence nesting site selection of Common Buzzards by considering predictors at the local scale (50m), the nesting territory scale (200m) as well as at the home range scale (1000m). To derive variables at the nesting territory and the home range scale, we calculated focal neighborhood means of the predictors for these scale-specific radii.

For the variable of habitat type, we used the Digital Landcover Model 2018 for Germany (LBM-DE 2018, available at <https://gdz.bkg.bund.de/index.php/default/digitales-landbedeckungsmodell-fur-deutschland-stand-2018-lbm-de2018.html>), aggregating the classes as followed: a) residential: all combinations of land cover and land use reflecting residential, industrial or administrative areas, b) cemeteries: land cover class B242 and land use N141 (urban cemeteries), c) city parks: all combinations of land cover and land use reflecting park and recreational areas, d) natural mixed: all combinations of land cover and land use reflecting predominantly impervious areas with little to no anthropogenically usage and medium to high levels of vegetation (e.g. forested edges of unused dumpsites, fallow land, orchards, cultivated pasture land with tree vegetation, e) urban mixed: all combinations of land cover and land use reflecting predominantly sealed and anthropogenically used areas with little vegetational cover, mostly along edge lines (e.g. roads with tree rows, landfills with edge vegetation etc.), f) others: all other combinations of land cover and land use classes.

We considered forest area (all combinations of land cover and land use reflecting coniferous or deciduous core forest areas of national parks or used forest) within the city area independently from the analysis, as it was not present within our study area (city core area).

We generated all predictors at a spatial resolution of 50m and projected all data to the WGS84 UTM Zone 33 coordinate system. We matched each species occurrence with the respective predictor from the closest year with available data for fitting the models. The variable imperviousness (200m) was highly correlated to tree cover density and therefore excluded from our final models.

**S6: Correlogram of the environmental predictors considered for the maxent model predicting habitat suitability for breeding of Common Buzzards.** Data includes observed nest sites (used, n = 225) and random background points (available, n = 2250). For variable pairs with Pearson's |r| > 0.7, the variables yielding superior model performance were retained.

**
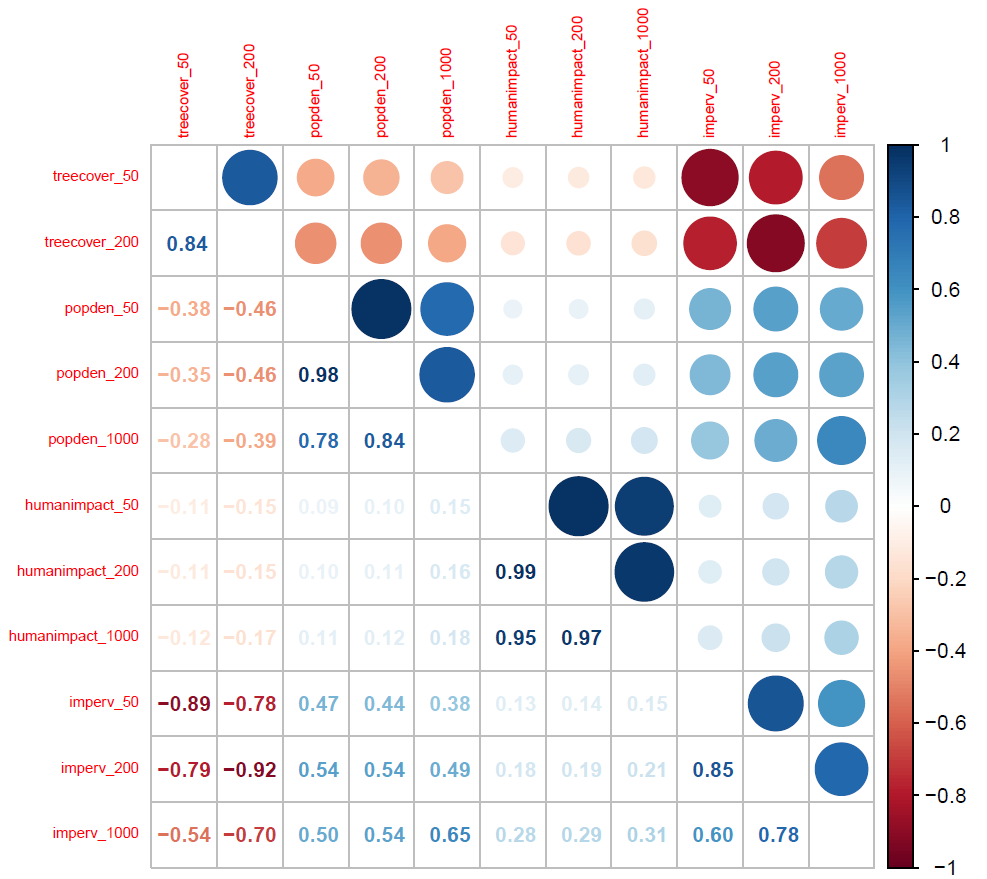
**

**S7: Variable distribution of the five environmental predictors used in the final maxent model predicting habitat suitability for breeding of Common Buzzards.** Data includes observed nest sites (used, n = 225) and random background points (available, n = 2250).

#
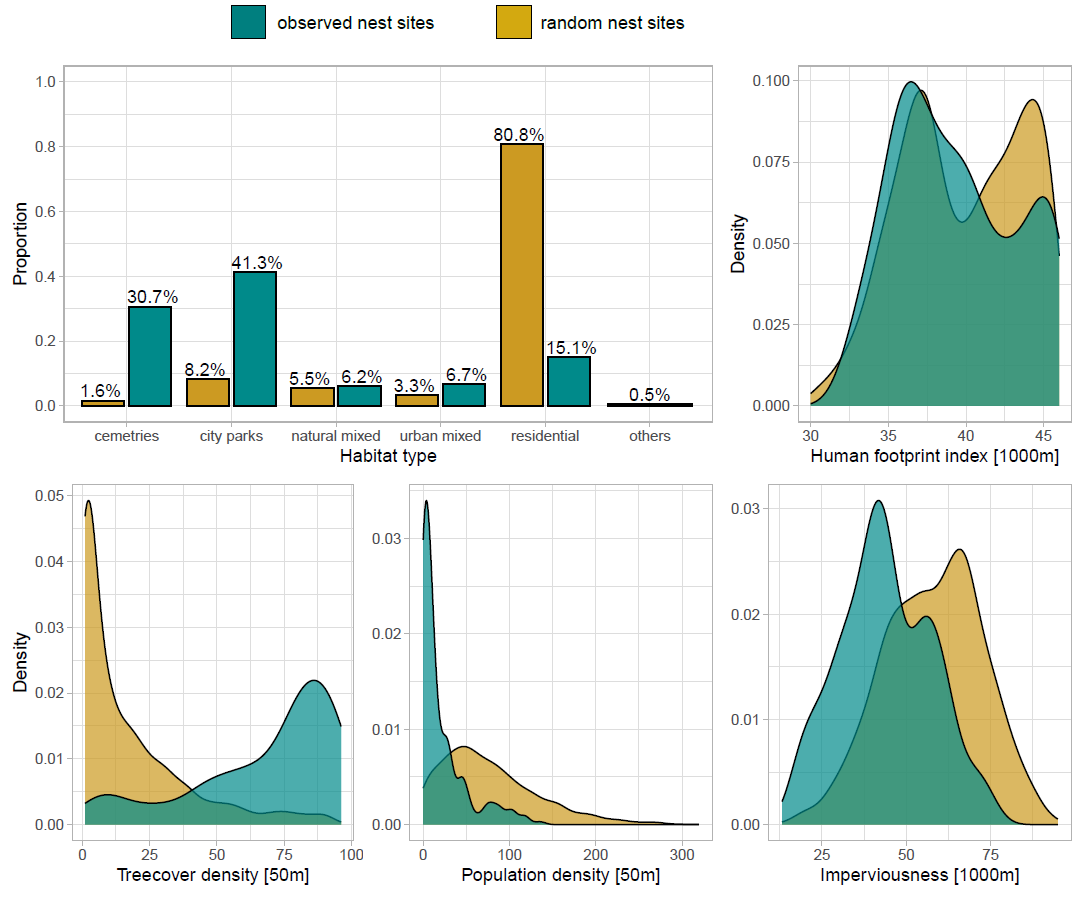


**S8: Parameter settings and performance metrics of maximum entropy (Maxent) models predicting suitability of breeding habitat for Common Buzzards in Berlin (Germany).** A 10-fold internal cross-validation was used to validate all models. Bold = final model used for continuous and binary predictions based on the highest AUC (Area Under the Curve) and TSS (True Skill Statistics).

| Features | Regularization | AUC | dAUC | TSS | dTSS |
| --- | --- | --- | --- | --- | --- |
| **Linear + quadratic + product + hinge** | **1** | **0.915** | **0** | **0.746** | **0** |
| Linear + quadratic + product + hinge | 2 | 0.912 | 0.003 | 0.745 | 0.001 |
| Linear + quadratic + product + hinge | 3 | 0.911 | 0.004 | 0.743 | 0.003 |
| Linear + quadratic + product + hinge | 4 | 0.911 | 0.004 | 0.744 | 0.002 |
| Linear + quadratic + product + hinge | 5 | 0.911 | 0.004 | 0.744 | 0.002 |
| Hinge | 1 | 0.914 | 0.001 | 0.745 | 0.001 |
| Hinge | 2 | 0.914 | 0.003 | 0.743 | 0.003 |
| Hinge | 3 | 0.912 | 0.003 | 0.745 | 0.001 |
| Hinge | 4 | 0.910 | 0.005 | 0.744 | 0.002 |
| Hinge | 5 | 0.910 | 0.005 | 0.744 | 0.002 |
| Hinge + product | 1 | 0.914 | 0.001 | 0.746 | 0 |
| Hinge + product | 2 | 0.912 | 0.003 | 0.746 | 0 |
| Hinge + product | 3 | 0.910 | 0.005 | 0.744 | 0.002 |
| Hinge + product | 4 | 0.911 | 0.004 | 0.742 | 0.004 |
| Hinge + product | 5 | 0.911 | 0.004 | 0.74 | 0.006 |

**S9: Partial dependency plots of the final habitat suitability model**. All predictor variables were considered in the final maxent model yielding the highest model performance, as measured by AUC.


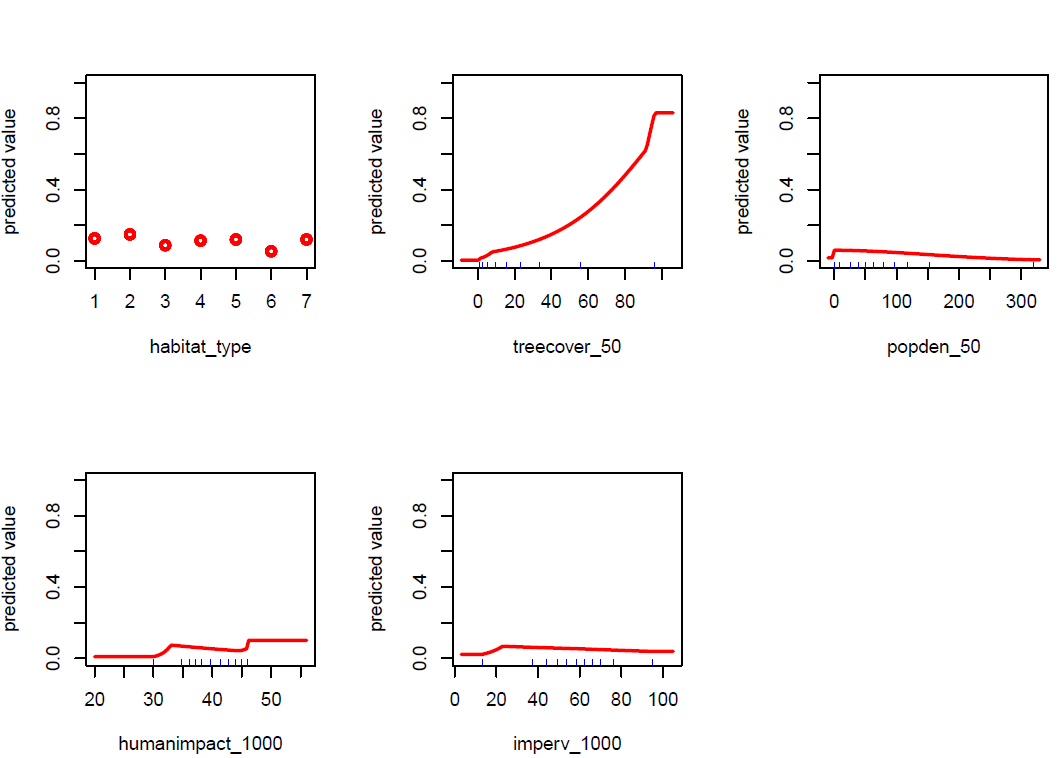


**S10: Total area contribution of potential environmentally suitable breeding habitat.** a) Within the study area (core city area). b) Within the total city area. Values were accumulated over all patches of the same habitat type. Habitats are ordered in ascending order along a gradient of high to low overall human impact.


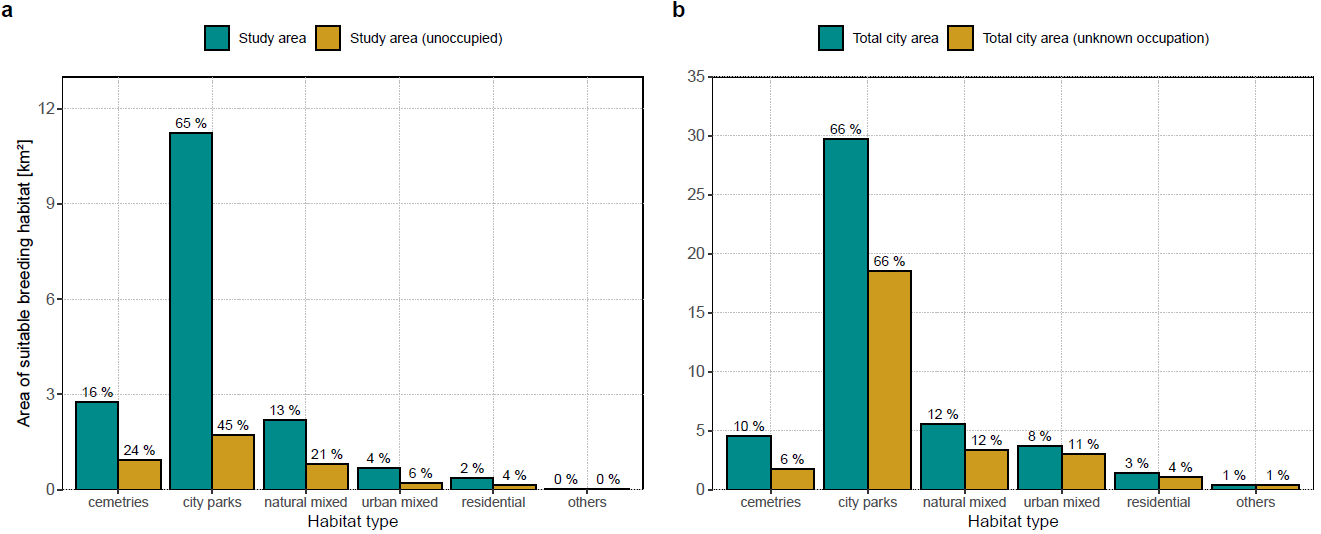

Supplement: Supplementary file 1 — Data S1: ece373097‐sup‐0001‐Supinfo.docx. [file ECE3-16-e73097-s001.docx]
